# Antiviral Surface Protection of Polyhedral Oligomeric Silsesquioxanes (POSS)–Appended Hybrid Surface Materials

**DOI:** 10.1155/ijbm/6543607

**Published:** 2026-06-19

**Authors:** Bibek Pramanik, Aparna Varma, Chandan Kumar Pal, Jakir Ahmed, Krishnendu Hazra, Totan Ghosh, Amirul Islam Mallick, Krishnendu Maji

**Affiliations:** ^1^ Department of Applied Chemistry, Maulana Abul Kalam Azad University of Technology (MAKAUT), Nadia, Haringhata, 741249, West Bengal, India; ^2^ Department of Biological Sciences and Centre for Advanced Functional Materials (CAFM), Indian Institutes of Science Education and Research Kolkata (IISER-K), Nadia, Mohanpur, 741246, West Bengal, India; ^3^ Department of Chemical Sciences, Indian Institutes of Science Education and Research Kolkata (IISER-K), Nadia, Mohanpur, 741246, West Bengal, India

## Abstract

Rapid technological advancement has led to a growing need for new materials with enhanced properties to meet the demands of emerging applications. Traditional single‐component materials often fail to meet the required demands, pushing the exploration of new composite materials. In these contexts, polyhedral oligomeric silsesquioxanes (POSS) as hybrid nanomaterials exhibit excellent mechanical, thermal and chemical stability, making them ideal for advanced material design. In addition, their hydrophobic nature and ability to be functionalized enable antimicrobial properties with enhanced resistance to microbial adhesion. Compared to hybrid nanomaterials, single‐component antiviral materials have limitations, including short‐term durability and low surface stability. The rationale for developing the POSS–stearic acid hybrid composite is based on the ability to form a rigid nanocage framework of POSS, which provides structural robustness and enhanced stability. We show that by combining POSS with stearic acid, the hybrid material displays greater hydrophobicity than its individual components, making it an effective surface coating material that prevents the attachment of microbial pathogens, including viruses, such as human coronavirus‐OC43 (HCoV‐OC43) and Type A influenza virus (H1N1). We provide evidence that the present material not only serves as a barrier to viral attachment to the surface but also significantly reduces viral infectivity, possibly through direct neutralization, thereby offering a promising strategy for mitigating surface‐mediated transmission of respiratory viruses.

## 1. Introduction

Recent advances in cutting‐edge polymer chemistry demand new materials with exceptional properties to meet the growing needs of emerging applications across various fields. Traditional single‐component molecules, while offering excellent properties, are often limited in their functional range. Thus, the idea of using composites, combining two or more distinct molecular entities to develop better‐performing materials, will be highly interesting [1]. In the past decades, silsesquioxane, an inorganic–organic hybrid material, has drawn significant interest in the fabrication of different versatile hybrid materials due to its inherent thermal, chemical and mechanical stability and flexibility [2–4]. Among the various types of silsesquioxanes, polyhedral oligomeric silsesquioxanes (POSS) have emerged as a significant modifier. In general, POSS possess a well‐defined, symmetrical nanocage structure with an interior containing inorganic silicon and oxygen atoms, and an exterior with varying alkyl groups [5, 6]. In recent times, researchers across the globe have developed several POSS‐conjugated hybrid molecules and shown that incorporation of POSS substantially enhances the tensile strength of the composite nanomaterials [7, 8]. Based on these properties, POSS‐based materials are being used for a wide range of applications, from mechanical reinforcement to fire protection. In terms of other modifications, incorporation of POSS into poly(vinylidene fluoride) was shown to improve the morphology, viscoelasticity and thermal stability [9]. A significant improvement in viscoelastic properties has also been reported for POSS‐styrene and polystyrene systems [10, 11]. Recently, it was demonstrated that POSS increase the thermal resistance and viscoelasticity of PMMA films [12]. Given that flame‐retardant behaviour is a very important feature of POSS materials, it was demonstrated that combining POSS with phosphorus‐modified PMMA produced composites with superior flame retardancy and thermal stability [13]. In particular, based on these versatile properties, POSS‐engineered epoxy resin has been successfully developed as a fire‐safety material [14]. Reports are also available suggesting that POSS‐functionalized graphene significantly reduced the flammability of epoxy resin [15]. Therefore, phosphinate–POSS blends have shown great promise for creating fire‐resistant PET fibres, integrating textile engineering with flame‐retardant chemistry [16–18].

Beyond thermal performance, POSS materials are attractive for their inherent hydrophobicity. For example, POSS–diphenylalanine surface material exhibits self‐cleaning, pollution‐protective and antibacterial properties, primarily because of its strong hydrophobic character [19]. To this end, POSS–fluorine block copolymers have been formulated as durable hydrophobic coatings [20], while UV‐curable coatings demonstrated enhanced hardness and water repellency using POSS‐containing materials [21]. Recent reports suggest that fluorosilicone POSS‐based polymer can effectively serve as a nonwetting surface material [22]. Contemporary studies show the potential of designing UV‐curable hydrophobic coating material based on EP‐POSS [23]. In addition, successful synthesis of self‐healable, ultrahydrophobic polyurethane–POSS hybrid coating materials has also been reported previously [24]. With this background, we hypothesize that the concept of surface hydrophobicity holds significant potential for developing effective protective coatings against various microbial pathogens.

Previous studies by Haldar et al. have demonstrated that POSS‐peptide materials serve as an effective coating against *Escherichia coli* [19]. POSS‐modified hybrid molecules are significantly hydrophobic in nature due to the presence of an alkyl group connected to a silicon atom on the exterior part [25–28]. Once the POSS molecule has been conjugated with stearic acid, the resultant hybrid material becomes more hydrophobic due to the added hydrophobicity of stearic acid, which originates from its long alkyl chain [29–31].

A key lesson from the recent pandemic is the importance of developing new protective coating materials to prevent the spread of harmful viruses, such as the HCoV or influenza virus. These coatings act as barriers that reduce the likelihood of surface contamination by viruses and, hence, limit their transmission. Thus, developing an antiviral coating helps minimize the risk of infection, providing an added layer of safety for individuals in public spaces or healthcare environments. Herein, we present a POSS‐based/stearic acid–based hybrid material (Compound 1) as a coating agent with strong water‐repellent properties that effectively prevent the attachment and transmission of human coronavirus‐OC43 (HCoV‐OC43) and human influenza type A/PR/8/1934 (H1N1) on its surface.

**FIGURE 1:**
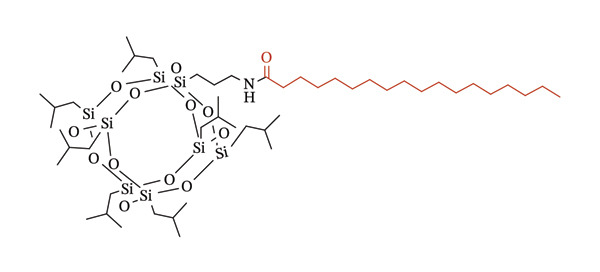
POSS‐based/stearic acid–based hybrid material (compound 1).

It is worth noting that, compared with commonly used hydrophobic antiviral coating materials, such as silicone‐based [32, 33] or fluoropolymer‐based [34] surface coating, the synthesis of POSS–stearic acid hybrid coatings does not rely on complex fabrication processes or specialized surface treatments. In contrast, the present coating approach combines the structural rigidity of POSS with the hydrophobicity of long‐chain fatty acids, enabling a solvent‐based process to achieve optimal antiviral effects. Collectively, we demonstrate a straightforward, one‐step DCC coupling‐based fabrication strategy to generate a dual‐hydrophobic molecular hybrid, offering a simpler, more accessible alternative to conventional multistep or surface‐engineered coating methods. Therefore, the present approach of POSS–stearic acid (Figure 1) hybridization can offer distinct advantages over conventional standalone hydrophobic materials by creating a stable dual‐hydrophobic molecular architecture. However, it is important to note that the observed effects are primarily attributed to antiviral surface protection and reduced viral attachment arising from enhanced surface hydrophobicity.

## 2. Materials and Methods

### 2.1. General Information

Solvents were dried using Na/benzophenone or calcium hydride before use. All chemicals were purchased and used as received unless otherwise mentioned. The ^1^H spectra were recorded on a BRUKER Avance 500 MHz and JEOL Advance 400 MHz spectrometer with residual undersaturated solvent as a reference. Record UV–Vis absorption spectra were recorded using a SHIMADZU Lab U‐19 spectrophotometer, and FT‐IR was recorded using SHIMADZU Lab Solutions IR equipment. Column chromatography was performed on neutral alumina and silica gel (Merck silica gel 100–200 mesh). For thin‐layer chromatography (TLC) analysis, Merck precoated TLC plates (silica gel 60 F254, 0.25 mm) were used. Visualization was accomplished by iodine chamber stain. Unless otherwise noted, all reactions were performed in oven‐dried glassware and carried out under open atmospheric conditions with magnetic stirring. Reaction temperatures are disclosed as the temperature of the bath surrounding the vessel unless otherwise mentioned.

#### 2.1.1. Synthesis of the Compound 1 (Scheme S1)

In an ice‐water bath, 247 mg stearic acid (0.87 mmol, 1.5 equiv.) was dissolved in 15 mL dry DCM. To this solution, 239 mg DCC (1.1 mmol, 2 equiv.) and 157 mg (1.16 mmol, 2 equiv.) HOBt were added, and the mixture was stirred for 10 min. To this mixture, 500 mg of POSS amine (0.58 mmol, 1 equiv.) was added, and the resulting reaction mixture was stirred for 24 h at room temperature. After the reaction was over, DCM was evaporated, and the residue was dissolved in 150 mL of ethyl acetate, and this organic layer was filtered. The ethyl acetate layer was washed with 50 mL of 2 M HCl (2 times) and 50 mL of 1 M sodium bicarbonate (2 times) consecutively. The organic layer was finally washed with 50 mL brine solution and dried over anhydrous sodium sulphate. The organic layer was evaporated, and the desired compound was purified using column chromatography with ethyl acetate as a solvent. The significant steric bulk of both reactants slightly lowers the reaction rate. Some material loss also occurs during filtration and purification. Due to the highly nonpolar nature of the product, it elutes rapidly in low‐polarity solvent systems (1%–2% ethyl acetate in hexane). The reaction is operationally straightforward and reproducible, with minimal synthetic challenges. About 342 mg pure compound (white solid) was obtained. Yield is 52%. Molecular Formulae: C_49_H_105_NO_13_Si_8_. ^1^H NMR (400 MHz, CDCl_3_, *δ* in ppm) 5.45 (s), 3.26 (d, *J* = 7.2 Hz), 2.16 (s), 1.86 (d, *J* = 6.0 Hz), 1.60 (s), 1.25 (s), 0.94 (dd, *J* = 24.5, 17.5 Hz), 0.60 (d, *J* = 7.3 Hz). ^13^C NMR (100 MHz, CDCl_3_) *δ* 173.96, 173.10, 77.41, 77.16, 76.91, 52.40, 50.64, 41.93, 36.73, 32.06, 29.84, 29.82, 29.79, 29.75, 29.63, 29.50, 29.47, 29.36, 25.73, 25.02, 24.93, 22.94, 22.83, 22.11, 14.26.^29^Si NMR (80 MHz, CDCl_3_) −67.61, −67.75, −67.85. Calculated mass 1139.5740, [M+H] + =1140.5867.

#### 2.1.2. Synthesis of POSS‐Phe (Scheme S2)

In an ice‐water bath, 230 mg Boc‐Phe‐OH (0.87 mmol, 1.5 equiv.) was dissolved in 15 mL dry DCM. About 239 mg DCC (1.1 mmol, 2 equiv.) and 157 mg (1.16 mmol, 2 equiv.) HOBt were added to the solution, and the mixture was stirred. After 10 min, 500 mg of POSS amine (0.58 mmol, 1 equiv.) was added to the reaction mixture, and the resulting reaction mixture was stirred for 24 h at room temperature. After the reaction was over, DCM was completely evaporated, and the residue was dissolved in 150 mL of ethyl acetate, and this organic layer was filtered. The ethyl acetate layer was washed with 50 mL of 2 M HCl for 2 times and 50 mL of 1 M sodium bicarbonate (2 times) consecutively. The organic layer was finally washed with 50 mL brine solution and dried over anhydrous sodium sulphate. The organic layer was evaporated to obtain a crude solid product, and the desired compound was purified using column chromatography with hexane‐ethyl acetate as a solvent. About 245 mg pure compound (POSS‐Phe, white solid) was obtained. Yield is 37.7%. Molecular formula: C_45_H_88_N_2_O_15_Si_8._^1^H NMR (400 MHz, CDCl_3_ Figure S7) *δ* 7.36 – 7.20 (m, 5H), 5.69 (s, 1H), 5.10 (s, 1H), 4.27 (q, *J* = 7.4 Hz, 1H), 3.33 – 2.87 (m, 4H), 1.87 (dh, *J* = 13.2, 6.6 Hz, 7H), 1.72 (s, 1H), 1.44 (s, 11H), 1.28 (s, 1H), 0.98 (d, *J* = 6.7 Hz, 41H), 0.62 (d, *J* = 7.0 Hz, 13H), 0.52 – 0.39 (m, 2H). ^13^C NMR (100 MHz, CDCl_3,_ Figure S8) *δ* 170.87, 136.87, 129.32, 128.68, 126.94, 77.34, 77.02, 76.70, 41.85, 38.81, 28.30, 26.92, 25.70, 25.68, 23.88, 23.85, 22.73, 22.50, 22.47, 9.29 (ESI): Mass calculated = 1120.4412, mass observed (Figure S9) [M+H]^+^:1121.4427.

#### 2.1.3. Synthesis of POSS‐Leu (Scheme S3)

In an ice‐water bath, Boc‐Leu‐OH (200 mg, 0.87 mmol, 1.5 equiv.) was dissolved in 15 mL of dry dichloromethane (DCM). To this solution, DCC (239 mg, 1.1 mmol, 2 equiv.) and HOBt (157 mg, 1.16 mmol, 2 equiv.) were added, and the reaction mixture was stirred for 10 min. Subsequently, POSS amine (500 mg, 0.58 mmol, 1 equiv.) was added, and the mixture was stirred at room temperature for 24 h. Upon completion of the reaction, the solvent was removed under reduced pressure, and the residue was dissolved in 150 mL of ethyl acetate. The organic layer was filtered and washed successively with 2 M HCl (50 mL × 2) and 1 M sodium bicarbonate solution (50 mL × 2). It was then washed with brine (50 mL) and dried over anhydrous sodium sulphate. The solvent was evaporated, and the crude product was purified by column chromatography using an ethyl acetate/hexane mixture as the eluent to obtain the desired compound (POSS‐Leu) as a white solid (326 mg). The yield is 51.7%. Molecular formula: C_42_H_90_N_2_O_15_Si_8_^1^H NMR (400 MHz, CDCl_3,_ Figure S10) *δ* 6.08 (t, *J* = 5.8 Hz, 1H), 4.85 (s, 1H), 4.03 (s, 1H), 3.23 (q, *J* = 6.7 Hz, 2H), 1.85 (dtd, *J* = 13.4, 6.7, 3.6 Hz, 7H), 1.64 – 1.52 (m, 3H), 1.47 (d, *J* = 8.4 Hz, 2H), 1.44 (s, 9H), 0.95 (d, *J* = 6.7 Hz, 48H), 0.60 (d, *J* = 7.0 Hz, 16H). ^13^C NMR (101 MHz, CDCl_3_ Figure S11) *δ* 172.25, 77.33, 77.02, 76.70, 41.81, 28.32, 25.70, 25.68, 24.78, 23.88, 23.85, 22.90, 22.50, 22.45, 22.10, 9.35. HRMS: Mass calculated:1086.4568, mass observed (ESI Figure S12) [M+H]^+^ = 1087.4583.

#### 2.1.4. Synthesis of Phe‐Stearic Acid (Same as Stearic Acid‐Phe‐OMe, Scheme S4)

In an ice‐water bath, 247 mg of stearic acid (0.87 mmol, 1.5 equiv.) was dissolved in 15 mL of dry DCM. To this solution, 239 mg DCC (1.1 mmol, 2 equiv.) and 157 mg (1.16 mmol, 2 equiv.) HOBt were added subsequently and stirred for 10 min. To this combination, 104 mg of H‐Phe‐OMe (0.58 mmol, 1 equiv.) was added, and the reaction mixture was allowed to stir at room temperature for 24 h. After the reaction was over, DCM was evaporated, and the residue was dissolved in 150 mL of ethyl acetate. The organic layer was filtered. The ethyl acetate layer was washed with 50 mL of 2 M HCl (2 times) and 50 mL of 1 M sodium bicarbonate (2 times), respectively. The organic layer was then washed with 50 mL of brine solution and dried over anhydrous sodium sulphate. The organic layer was evaporated, and the required compound was purified using column chromatography using hexane‐ethyl acetate as a solvent. About 118 mg of pure compound was obtained as a white solid. The yield is 45.6%. Molecular formula: C_28_H_47_NO_3._^1^H NMR (400 MHz, CDCl_3,_ Figure S13) *δ* 8.03 (d, *J* = 8.4 Hz, 1H), 7.67 (d, *J* = 8.3 Hz, 1H), 7.56 (t, *J* = 7.6 Hz, 1H), 7.42 (t, *J* = 7.7 Hz, 1H), 7.08 (d, *J* = 7.1 Hz, 1H), 6.22 (s, 2H), 5.89 (d, *J* = 7.9 Hz, 1H), 4.90 (q, *J* = 6.3 Hz, 1H), 3.72 (s, 3H), 3.12 (qd, *J* = 13.9, 5.9 Hz, 2H), 2.16 (t, *J* = 7.6 Hz, 2H), 1.84 (tt, *J* = 14.2, 7.1 Hz, 2H), 1.60 (dp, *J* = 21.6, 7.9 Hz, 3H), 1.25 (s, 17H), 0.95 (d, *J* = 6.6 Hz, 5H), 0.87 (t, *J* = 6.6 Hz, 4H), 0.60 (d, *J* = 7.0 Hz, 1H). ^13^C NMR (101 MHz, CDCl_3,_ Figure S14) *δ* 172.74, 172.22, 143.43, 135.91, 129.32, 129.26, 128.77, 128.71, 128.67, 128.55, 127.11, 125.05, 120.20, 109.41, 85.08, 77.36, 77.05, 76.73, 52.92, 52.30, 37.93, 36.57, 31.93, 29.70, 29.66, 29.62, 29.48, 29.36, 29.33, 29.21, 28.29, 25.70, 25.68, 25.56, 23.88, 23.85, 22.69, 22.50, 22.46, 14.12. HRMS (ESI): Mass calculated: 445.3629, mass observed (Figure S15) [M+H]^+^: 446.3641.

#### 2.1.5. Synthesis of Leu‐Stearic Acid (Same as Stearic Acid‐Leu‐OMe Scheme S5)

In an ice‐water bath, stearic acid (247 mg, 0.87 mmol, 1.5 equiv.) was dissolved in 15 mL of dry dichloromethane (DCM). To this solution, DCC (239 mg, 1.1 mmol, 2 equiv.) and HOBt (157 mg, 1.16 mmol, 2 equiv.) were added, and the mixture was stirred for 10 min. Subsequently, H‐Leu‐OMe (100 mg, 0.58 mmol, 1 equiv.) was introduced, and the reaction mixture was stirred at room temperature for 24 h. After completion of the reaction, the solvent was removed under reduced pressure, and the residue was dissolved in 150 mL of ethyl acetate. The organic layer was filtered and then washed sequentially with 2 M HCl (50 mL × 2) and 1 M sodium bicarbonate solution (50 mL × 2). It was further washed with brine (50 mL) and dried over anhydrous sodium sulphate. The solvent was evaporated, and the crude product was purified by column chromatography using an ethyl acetate/hexane mixture as the eluent to afford the desired compound as a white solid (130 mg). Yield is 54%. C_25_H_49_NO_3_. ^1^H NMR (500 MHz, CDCl_3_ (Figure S16), *δ* 5.83 (d, *J* = 8.4 Hz, 1H), 4.65 (td, *J* = 8.7, 4.2 Hz, 1H), 3.72 (s, 3H), 2.20 (t, *J* = 7.3 Hz, 2H), 1.64 (d, *J* = 5.9 Hz, 3H), 1.52 (q, *J* = 8.9 Hz, 1H), 1.24 (s, 20H), 0.94 (d, *J* = 4.9 Hz, 5H), 0.87 (t, *J* = 6.4 Hz, 3H). ^13^C NMR (126 MHz, CDCl_3_ (Figure S17) *δ* 173.96, 173.10, 77.41, 77.16, 76.91, 52.40, 50.64, 41.93, 36.73, 32.06, 29.84, 29.82, 29.79, 29.75, 29.63, 29.50, 29.47, 29.36, 25.73, 25.02, 24.93, 22.94, 22.83, 22.11, 14.26. HRMS (ESI): Mass calculated = 411.3785, mass observed (Figure S18) [M+H] ^+^: 412.3787.

### 2.2. Cells and Viruses

MDCK and Vero E6 cells (NCCS, Pune, India) were grown and maintained in Dulbecco’s modified Eagle’s medium (DMEM; Gibco, USA) with supplementation of 10% foetal bovine serum (Gibco, USA), 100 U/mL penicillin and 100 μg/mL streptomycin (1% P/S) (Gibco, USA) at 37°C under 5% CO_2_ supply. The influenza type A/PR/8/1934 (H1N1) virus stock was obtained from ATCC (ATCCVR95) and grown in the allantoic fluid of embryonated chicken eggs (∼10–11 days old) following previously published protocols. The harvested virus was aliquoted, stored and used to infect MDCK cells in DMEM supplemented with 1% BSA and 1% P/S. For the infection study, DMEM media supplemented with 0.5% BSA, 1 μg/mL TPCK‐treated trypsin and 1% P/S were used as infection media for the H1N1 virus, as applicable.

The HCoV‐OC43 was obtained from BEI Resources (NR‐52725, NIAID, NIH, USA) and was grown and maintained in Vero E6 cells. HCoV‐OC43 virus was grown and maintained in DMEM supplemented with 2% FBS and 1% P/S, as per published protocols. This medium is used as the infection medium for virus infection studies wherever applicable. The harvested virus‐containing medium was analysed for both viruses and infectivity titre, with TCID50/mL determined in MDCK and Vero E6 cells, respectively, as per previously published protocols [35, 36]. The details of cell data (Species, Tissue of Origin, Official Cell Line Name) have been mentioned at point number five in this main manuscript.

### 2.3. Preparation of POSS‐Coated Material for Antiviral Assessment

**FIGURE 2:**
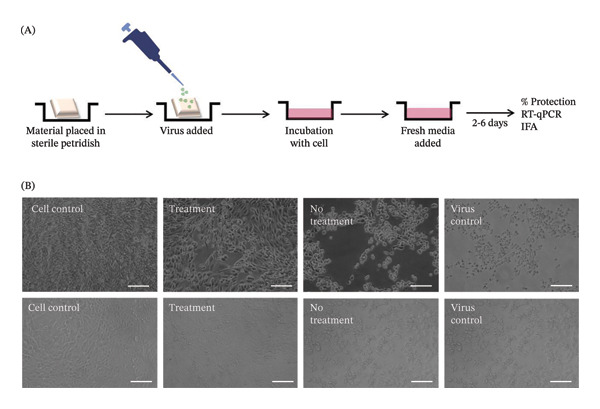
(A) A schematic of the experimental workflow. (B) Reduction in the cytopathic effects of cells after POSS exposure of cells charged with (A/PR/8/1934/H1N1) and human coronavirus (HCoV‐OC43), characterized by rounding, detachment and death, indicating a significant in vitro cell protection. Panel 1: CPE effect in MDCK cells after H1N1 virus infection after POSS coating. Panel 2: CPE effect of Vero E6 cells after HCoV‐OC43 infection after POSS coating.

A dilute solution of the materials (10 mg/1.5 mL) in DCM was drop‐cast onto the rough surface of a butter paper and left to air‐dry completely under sterile conditions. Next, a small portion (2 cm × 2 cm) was excised aseptically with sterilized scissors and carefully placed in a 35‐mm petri dish. Next, the cut portions were evenly speared with the respective virus stocks (1 MOI of H1N1 virus and 200 TCID50/mL of HCoV‐OC43 virus) separately. This was left undisturbed for 5 min and then rinsed thoroughly with the respective infection medium for 3–4 times to ensure that all residual viral particles were collected in the medium. This medium was then charged to the respective cell lines and proceeded for further assessments. A schematic of this experimental workflow is presented in Figure 2A. Additionally, the reason behind choosing butter paper was given as follows. Standard A4 cellulose paper is highly hydrophilic, and during preliminary tests, the drop‐cast solution was absorbed almost instantaneously, preventing effective film formation and making control experiments challenging. In contrast, butter paper possesses a relatively rough, hydrophobic surface with lower absorptivity. This rougher side facilitates controlled solvent evaporation, enabling the formation of more uniform and reproducible coatings. Therefore, butter paper was found to be a more suitable substrate for easy and consistent coating.

### 2.4. Assessment of Percentage Cell Viability After Viral Inhibition

To assess the percentage of cell viability by the residual viral particles after POSS coating, we grew the respective cells (MDCK and Vero E6 cells) until confluency (1 × 10^6^ cells per well) in a standard 12‐well plate (Tarsons, India) at 37°C under 5% CO_2_ supplementation. The media with the residual viral particles were then charged to the cells and incubated for the respective time points, as mentioned earlier. The cells were then observed to develop visible cytopathic effects (CPEs) and imaged using a Nikon Eclipse TS 100 light microscope at 20X magnification. Next, the media were discarded, washed with 1X PBS and proceeded further for a standard 3‐[4,5‐dimethyl thiazol‐2‐yl]‐2,5‐diphenyl tetrazolium bromide (MTT) assay to calculate the percentage of cell viability after virus infection, as per previously published protocols [36, 37]. The percentage of cell protection was further determined using the following formula:

(1) % protection=A−BC−B×100,

where A is the absorbance value of residual virus‐infected cells, B is the absorbance of untreated virus‐infected cells (positive control), and C is the absorbance of uninfected cells (negative control).

### 2.5. Viral Detection by Indirect Immunofluorescence Assay (IFA)

To detect residual virus after treatment, the respective cells were grown in 6‐well tissue culture plates with coverslips until ∼70%‐80% confluency and subsequently incubated in media containing the residual viral particles (as mentioned earlier) for the respective time points to monitor the onset of CPEs. Next, the cells were washed with 1X PBS, fixed with 4% paraformaldehyde (PFA) at room temperature for 15 min, treated with ice‐cold permeabilization buffer (1% Triton‐X in 1X PBS) for 30 min at 4°C and blocked with 1% BSA solution with 0.05% Tween‐20 for 1 h at room temperature. The cells were then incubated with the 1:1000 dilution of the respective primary antibody (i.e. mouse monoclonal Influenza A NP antibody (Santa Cruz Biotechnology, USA) for nucleoprotein H1N1 virus and rabbit polyclonal coronavirus Nucleocapsid antibody (Sino Biologicals, Japan) for nucleoprotein of HCoV‐OC43 at 4°C overnight. After the incubation, the cells were washed thrice with 1X PBS and then probed with the respective FITC‐labelled IgG secondary antibody (Fab_2_, 1:1000; Thermo Fisher Scientific) for 2 h at room temperature. After thorough washing with 1X PBS, the cells were then counter‐stained with 4′,6‐diamidino‐2‐phenylindole‐dihydrochloride (DAPI) and mounted with VECTASHIELD mounting media (Vector Laboratories, USA). The imaging was performed using the Leica TCS SP8 confocal microscope under 63X magnification and processing using ImageJ software.

### 2.6. Quantification of Viral *M* and N Gene Copy Number by RT‐qPCR

To assess the impact of POSS coating on viral infectivity and replication, we quantified viral transcription in infected cells by qRT‐PCR. Briefly, 1000 ng of total RNA extracted from the respective cells after the respective virus infections were used for cDNA synthesis with the iScript cDNA synthesis kit from Bio‐Rad, USA, according to the manufacturer’s protocol. Next, a 1:2 dilution of the respective cDNA was then used for the quantitative real‐time PCR (RT‐qPCR) analysis using the 2X SYBR green PCR master mix (Applied Biosystems, USA) with *M* gene‐specific primer sets (for H1N1 virus) or N gene‐specific primer sets (for HCoV‐OC43 virus) according to the manufacturer’s protocol in CFX96 real‐time PCR system (Bio‐Rad, USA) to obtain the respective Ct values. The cycling conditions were 95°C for 2 min (one cycle), 95°C for 15 s and 60°C for 1 min (40 cycles). The Ct values were then converted into respective gene transcript numbers (*n*_molecules_) using the previously generated influenza *M* (242 bp) and HCoV OC43 N gene (188 bp) specific standard by using the following equation:

(2) nmolecules=mtemplate X NAk X Nbases X 109,

where *m*_template _ [ng] is the amount of pMD20‐M gene recombinant plasmid (Promega, USA) for H1N1 virus or pGEM‐T‐N recombinant plasmid (Promega, USA) for HCoV‐OC43,  *N*_bases_ [bp] is the length of the viral gene (bp), *k* is the average mass of one base, that is ∼340 [Da/bp], and  *N*_*A*_ is the Avogadro constant [mol^−1^] [36, 38]. Viral transcript quantification was performed using an absolute quantification approach based on standard curves. Therefore, normalization to host housekeeping genes was not applied.

### 2.7. Assessment of Viral Neutralization

To verify the neutralizing effect of the POSS compound on the viral particles, we incubated 2 μL of POSS compound (∼188 mg/mL in DCM) with the respective viral particles (1 MOI H1N1 and 200 TCID50 HCoV‐OC43) in the respective infection media for 1 h at 37°C. After the incubation, the neutralized viral particles were charged to the respective cells (MDCK cells for H1N1 and Vero E6 cells for HCoV‐OC43) for 2 h and 3 h, respectively. Next, the media were changed, washed with 1X PBS, replenished with fresh infection media and incubated for 2 days in case of H1N1 virus and ∼5–6 days for HCoV‐OC43 infection before proceeding to standard MTT and IFA analysis, as described in Sections 2.5 and 2.6.

### 2.8. Statistical Analysis

The graphs were plotted and analysed using the GraphPad Prism statistical software Version 8.4.2 (USA). The data for the percentage protection were analysed using the unpaired Student’s *t*‐test with Welch’s correction. The data obtained from the RT‐qPCR were analysed using the Student’s *t*‐test (two‐tailed, unpaired) to compare differences among the experimental groups. The normality of each data set was checked using the Shapiro–Wilk test.

## 3. Results and Discussion

### 3.1. Synthesis and Characterization of POSS‐Appended Hybrid Material (Compound 1)

The compound 1 has been synthesized by solution‐phase standard methodology using POSS amine and stearic acid and DCC as coupling agents. The compound 1 has been characterized by IR spectroscopy (shown in Figure S1), ^1^H NMR (Figure S3), ^13^C NMR (Figure S4), ^29^Si NMR (Figure S5) and mass spectroscopy (Figure S6). The ^1^H NMR, mass spectrometry and IR data confirm the synthesis of the compound. FT‐IR spectroscopy exhibited (Figure S1) a prominent band at 1114 cm^−1^, attributed to Si‐O‐Si linkage [39]. The peaks appearing in the region of 1642 cm^−1^ are due to the amide group. The intensity of the amide band is significantly lower compared to the Si‐O‐Si band. This is because there is a single amide linkage compared to the multiple Si‐O‐Si linkages in a molecule. The appearance of an amide linkage, as indicated by the IR, indicates the attachment of stearic acid to the POSS amine.

### 3.2. Hydrophobicity Study

**FIGURE 3:**
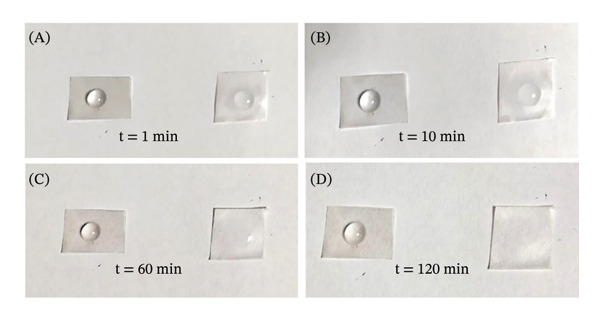
Hydrophobicity study of the coated surface at different time intervals. (A), (B), (C), (D).

The molecule demonstrated notable hydrophobic properties due to several hydrophobic components, including isopropyl groups attached to a silicon atom and a long alkyl chain from stearic acid. To test hydrophobicity, a water droplet was placed on the coated paper surface (with a loading of 0.025 mg/cm^2^), and a control experiment was conducted by placing a water droplet on an uncoated paper surface. On the coated surface, the water droplet maintained an almost spherical shape for up to 2 h. In contrast, on the uncoated surface, the water droplet was quickly absorbed, indicating the significant hydrophobic nature of the molecule (shown in Figure 3). The contact angle was ∼ 113° (Figure S2). To understand the surface morphology, FE‐SEM of the coated surface was carried out. FE‐SEM data showed smooth surface morphology (shown in Figure S19). The sample was prepared in a similar way as mentioned in Section 2.4.

### 3.3. POSS Coating Reduced Human H1N1 and HCoV‐OC43 Virus Viral Infectivity

#### 3.3.1. Reduction in the Cell Cytopathic Effects (CPEs) After Surface Coating of POSS Compound

To study the effect of surface coating on viral infectivity, we observed almost 60%–70% protection of both MDCK and Vero E6 cells from H1N1 and HCoV‐OC43 virus infection, respectively, as observed by a marked difference in the CPEs indicated by the characteristic features, such as shrinkage, cell rounding, detachment and death (Figure 2B).

#### 3.3.2. POSS Treatment Provided Enhanced Cell Protection Against the Virus

**FIGURE 4:**
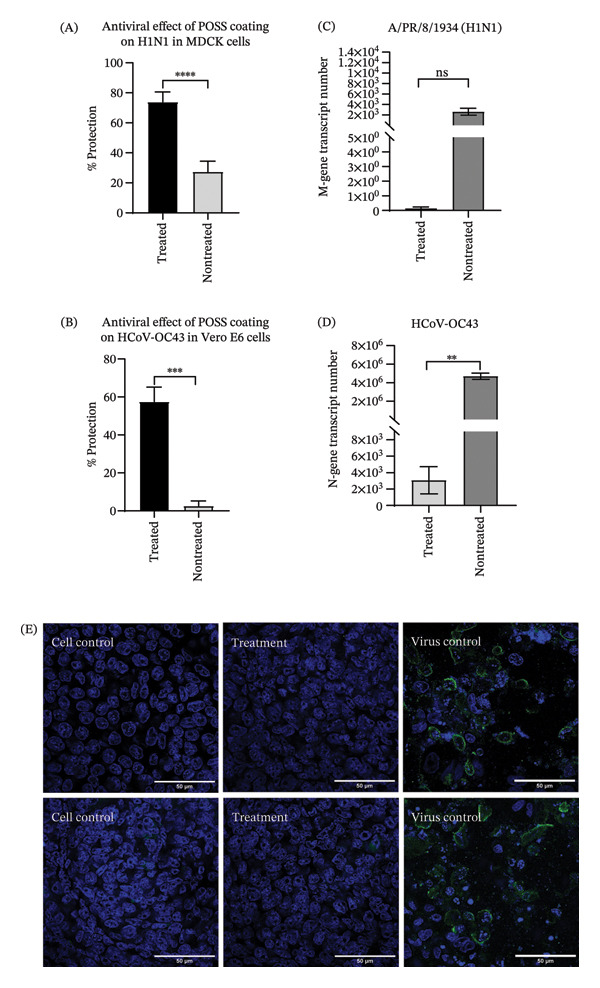
Effect of POSS coating on MDCK and Vero E6 cell viability and reduced viral replication against influenza H1N1 virus and HCoV‐OC43. (A) Percentage protection of MDCK cells after POSS coating. (B) Percentage protection of Vero E6 cells after POSS coating. Each bar indicates the gene transcript number ± SE of four independent experiments, and asterisks (^∗^) denote statistically significant differences (^∗^ *p* ≤ 0.05) in comparison with the control (virus‐only) group. (C) and (D) Quantifying viral M and N gene transcript numbers, respectively, in MDCK and Vero E6 cells determined by RT‐qPCR. Each bar indicates the gene transcript number ± SE of three independent experiments. Asterisks (^∗^) denote statistically significant differences (^∗^ *p* ≤ 0.05) in comparison with the control (virus‐only) group. (E) The reduction in accumulation of influenza‐NP proteins after POSS exposure was confirmed by IFA. Green fluorescence corresponds to viral NP protein, and blue fluorescence corresponds to DAPI nuclear staining.

**FIGURE 5:**
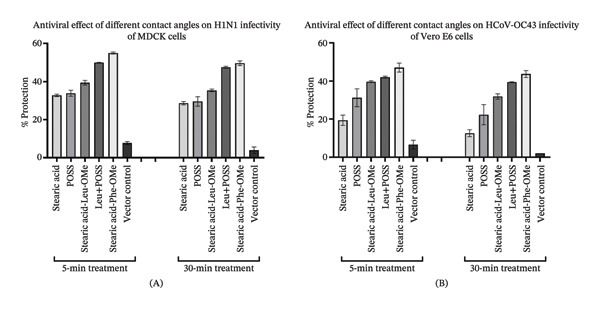
Effect of exposure duration and surface hydrophobicity on viral infectivity following contact with coated materials. The influenza/H1N1 and HCoV‐OC43 viruses were exposed to surfaces coated with materials of varying hydrophobicity (stearic acid, POSS and their hybrid conjugates) for defined time intervals (5 min and 30 min). The residual virus collected after surface exposure was subsequently used to infect (A) MDCK cells (H1N1) and (B) Vero E6 cells (HCoV‐OC43), and the percentage cell protection was calculated to determine the residual virus infectivity following exposure to the respective surfaces. A marked reduction in the viral infectivity was observed, with more hydrophobic coatings exhibiting greater antiviral effects at both exposure durations. Vector control represents virus exposed to uncoated butter paper, serving as a surface control and showing infectivity comparable to virus control. Data are represented as mean ± SE from three independent experiments.

Incubating the viruses with the POSS‐coated surface for a short duration (5 min) effectively protected the cells from virus infection. In the case of the H1N1 virus, the cell survivability was ∼60%, whereas for the HCoV‐OC43 virus, cell survivability was ∼70% (Figure 4A, B). In addition, to evaluate the antiviral efficacy as a function of exposure time, we checked the residual virus infectivity following 30‐min contact of the virus sample with the coated surfaces. While a modest reduction in infectivity for both H1N1 and HCoV‐OC43 viruses was observed at 30 min compared to 5 min, the antiviral activity remained highly significant at both time points (Figure 5). These results suggest that the POSS–stearic acid hybrid surface coating provides sustained antiviral protection over time.

#### 3.3.3. Reduction in Intracellular Accumulation of Viral Protein

To further confirm the protective efficacy of POSS coating, an indirect IFA was performed to detect viral nucleoproteins by infecting cells with a POSS‐treated virus. We observed that cells charged with virus‐containing medium (washing from the coated surface) show significantly lower intracellular protein levels than cells charged with medium collected from the uncoated surface (Figure 4E).

#### 3.3.4. Reduction in the Viral Transcript Numbers After POSS Coating

To determine the effect of POSS coating on viral infectivity in infected cells, qRT‐PCR was performed to quantify the viral *M* gene (for H1N1 virus) and N gene (for HCoV‐OC43) transcript levels. Compared with the respective standard curves, a modest reduction in viral gene transcript levels (*p* = 0.0573 for human H1N1; *p* = 0.0052 for HCoV‐OC43) was observed in both cases relative to the vector control (Figure 4C, D). Although this reduction does not meet the conventional threshold for statistical significance, it suggests a trend towards reduced viral infectivity.

### 3.4. POSS Effectively Neutralize Human H1N1 and HCoV‐OC43 Virus

#### 3.4.1. Enhanced Cell Protection After Viral Neutralization

**FIGURE 6:**
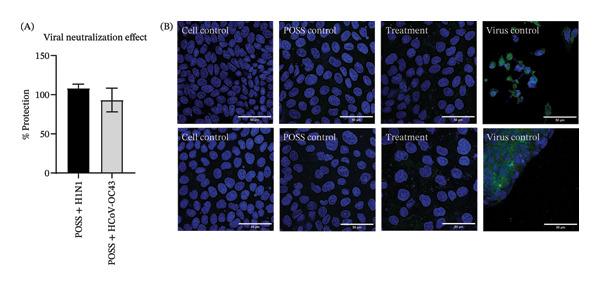
Neutralization effect of POSS on cell viability and viral protein accumulation. (A) Percentage protection of MDCK cells (in case of H1N1 virus) and Vero E6 cells (in case of HCoV‐OC43) after POSS neutralization. Each bar indicates the percentage cell viability ± SE of three independent experiments. (B) Visualization of viral NP proteins after virus neutralization confirmed by immunofluorescence imaging using FITC and DAPI filters. Green fluorescence corresponds to viral NP protein, and blue fluorescence corresponds to DAPI nuclear staining.

Viral neutralization with the POSS compound showed very promising protection of the cells from viral infection. The POSS compound successfully neutralized both MDCK and Vero E6 cells infected with H1N1 and HCoV‐OC43, respectively, and provided nearly absolute protection (Figure 6A).

#### 3.4.2. Reduced Accumulation of Viral Proteins After Virus Neutralization

In support of the findings of the MTT assay, IFA performed against the nucleoprotein of H1N1 and nucleocapsid protein of HCoV‐OC43 showed a similar significant reduction in viral protein accumulation after neutralization, providing almost absolute protection of MDCK and Vero E6 cells, respectively (Figure 6B). As the MTT assay reflects cell viability and serves as an indirect measure of viral infectivity through virus‐induced CPEs, the findings were further supported by both IFA and RT‐qPCR analyses.

### 3.5. Discussion

The study demonstrates the development of POSS‐appended stearic acid–based coating materials which are capable of preventing attachment and subsequent infectivity of HCoV‐OC43 and type A influenza virus (H1N1). The distinct feature of this study lies in the synthesis and application of the POSS–stearic acid hybrid, which enhances surface hydrophobicity and provides a potent barrier against virus attachment. This approach not only provides a much‐needed solution for controlling viral spread but also addresses the critical issue of surface contamination in various public and healthcare environments.

The synthesis, confirmed by ^1^H NMR and other analytical techniques, demonstrates the successful incorporation of POSS into stearic acid, yielding a hybrid material with significantly increased hydrophobicity. The observed contact angle measurements further support this, showing a marked difference in water droplet behaviour between coated and uncoated surfaces. Moreover, the addition of stearic acid to the POSS molecule resulted in a material that repelled water more effectively, thereby hindering viral particles from adhering to the coated surfaces.

The comparison between coated and uncoated surfaces revealed that the hydrophobic coating effectively prevented water droplet absorption, underscoring its capability to create a physical barrier that reduces viral adherence. Such hydrophobic coatings are thus essential for mitigating the risk of pathogen transmission via surface contact, a major route for viruses, such as HCoV‐OC43 and H1N1. The antiviral surface protection or reduced viral attachment of the POSS‐based/stearic acid–based hybrid material was systematically evaluated through in vitro experiments. The in vitro experiments using the MDCK and Vero E6 cells demonstrated a significant reduction in viral infectivity following exposure to H1N1 and HCoV‐OC43 viruses. In terms of cell viability, we observed nearly 60%–70% reduction in virus‐induced CPEs in MDCK and Vero E6 cells (as shown in Figure 2B). Therefore, we propose that the dual function of the POSS‐based/stearic acid–based hybrid materials not only acts as a barrier to viral attachment but also appears to interfere with viral survival, leading to a low number of active viruses that can infect host cells. The observed reduction in viral protein accumulation and transcript levels further supports the hypothesis that the coating inhibits viral replication after attachment, effectively neutralizing the virus at multiple stages. The ability of the coating to protect cells from viral infection, even with a brief exposure time of 5 min, indicates that the material is not only effective but also fast‐acting. To establish a quantitative correlation between surface hydrophobicity and antiviral efficacy, we systematically evaluated multiple coating formulations with varying compositions, including POSS, stearic acid and their hybrid conjugates (shown in Figure 5). The synthesis of these derivatives has been described under Section 2.1. These materials exhibit different hydrophobicity (due to different hydrophobic components), which corresponds to different antiviral performance. Notably, coatings with higher hydrophobic character (as composed of more hydrophobic components), POSS–stearic acid hybrid, demonstrated significantly improved cell protection compared to other components, such as stearic acid or POSS alone, or stearic acid‐Leu‐OMe (named as Leu‐stearic acid also) and stearic acid‐Phe‐OMe (named as Phe‐stearic acid also), POSS‐Phe and POSS‐Leu. A strong positive correlation was observed following percentage cell protection, suggesting that hydrophobicity effectively suppresses viral attachment and subsequent infectivity. Collectively, these findings support the hypothesis that surface hydrophobicity plays a pivotal role in inhibiting viral adsorption, thereby enhancing antiviral efficacy.

Furthermore, we confirmed that the increasing hydrophobicity of the coated surfaces is directly correlated with antiviral efficacy over time, reinforcing the role of surface hydrophobicity in reducing viral adherence. Together, we demonstrate that the coating is not only rapidly effective but also retains its antiviral function over extended exposure durations (Figure 5).

The versatility of the POSS–stearic acid coating is also evident from its ability to neutralize both H1N1 and HCoV‐OC43 viruses, highlighting its broad‐spectrum antiviral properties. This property is essential for real‐world applications, where rapid antiviral surfaces could be crucial in high‐traffic environments, such as hospitals, public transportation and healthcare systems. The effectiveness of the coating materials in neutralizing viral activity also extends to a significant reduction in viral protein accumulation, further reinforcing their potential as protective coating materials. While these hybrid material–based hydrophobic coatings can reduce viral adhesion, the precise mechanisms underlying antiviral activity often remain unclear and require detailed investigation. While plaque assays are considered the gold standard for quantifying infectious virus, this study relies on complementary approaches, including MTT, IFA and RT‐qPCR, which collectively indicate reduced viral infectivity. Additional evidence from viral plaque assays would further strengthen the findings and will be considered in future studies. Moreover, the precise molecular mechanism remains to be fully elucidated to provide deeper insight into the mechanistic basis of the observed effects. Nevertheless, the demonstrated functional neutralization of the virus indicates near‐complete protection of host cells, accompanied by reduced viral protein accumulation and overall cell CPEs, thereby supporting a significant reduction in viral infectivity.

## 4. Conclusion

In conclusion, the POSS‐based/stearic acid–based hybrid material presented in this study offers a promising approach in controlling viral transmission. The present material with its enhanced hydrophobicity effectively prevents the attachment of HCoV‐OC43 and type A influenza virus (H1N1) to its surface and significantly reduces their transmission. This study highlights the dual function of the POSS‐based/stearic acid–based hybrid materials: Not only do they act as a barrier to viral attachment, but they also appear to significantly reduce viral attachment and infectivity, including through a direct neutralization effect. This strategy offers an effective means to limit viral transmission while simultaneously tackling the persistent challenge of surface contamination in public and healthcare settings.

## 5. Species, Tissue of Origin and Official Cell Line Name

### 5.1. MDCK Cells

Species: *Canis lupus familiaris* (Dog).

Sex: Female.

Tissue of origin: Kidney.

Official cell line Name: Madin‐Darby Canine Kidney (MDCK).

### 5.2. Vero E6 Cells

Species: *Chlorocebus sabaeus* (African Green Monkey).

Sex: Female.

Tissue of origin: Kidney.

Official cell line name: Vero E6 (Vero C1008).

### 5.3. Source/Supplier and Date of Acquisition

Both MDCK and Vero E6 cell lines were procured from the National Centre for Cell Science (NCCS) cell repository, Pune, India.

### 5.4. Authentication Status

Both cell lines were authenticated at the time of procurement from NCCS, Pune, India. The match profile showed a 100% similarity with the reference profile. As these cell lines were authenticated upon acquisition, no additional authentication was performed for this study.

There are no reports of misidentification or contamination associated with this cell line.

### 5.5. Details and Rationale of Cell Line Usage

Vero E6 Cells (for HCoV‐OC43): Vero E6 cells, derived from African green monkey kidney epithelial tissue (female origin), are highly permissive to coronavirus infection and propagation of HCoVs, including OC43. Vero E6 cells develop clear CPE upon OC43 infection and are widely used for antiviral studies [40–42].

MDCK Cells (for Influenza A/H1N1): MDCK cells are derived from female canine kidney epithelial tissue and are considered the gold standard for influenza virus studies. They naturally express both *α* 2,3‐linked and *α* 2,6‐linked sialic acid receptors, which mediate influenza A virus entry, making them highly permissive to H1N1 infection. MDCK cells are routinely used for influenza virus isolation, titration and antiviral testing, and develop prominent CPE with high viral yields [42–45].

## Author Contributions

Bibek Pramanik and Aparna Varma contribute equally to the manuscript. Bibek Pramanik and Krishnendu Hazra have synthesized and characterized the compounds. Aparna Varma has conducted the biological experiments under the supervision of Amirul Islam Mallick.

Totan Ghosh, Chandan Kumar Pal and Krishnendu Maji have analysed the data. Jakir Ahmed has performed the ^1^H, ^13^C and ^29^Si (for compound 1) of all the newly synthesized compounds. Krishnendu Maji and Amirul Islam Mallick wrote, edited, and reviewed the manuscript.

## Funding

Krishnendu Maji acknowledges the Anusandhan National Research Foundation (ANRF) for financial support (Grant No. SRG/CS/2023/000841). Amirul Islam Mallick acknowledges the IISER Kolkata Institutional Fund (Academic Research Fund).

## Disclosure

All authors have approved the final version of the manuscript.

## Ethics Statement

The use of embryonated chicken eggs for virus propagation was approved by the Institute Animal Ethics Committee (IAEC), in accordance with the guidelines of the Committee for the Control and Supervision of Experiments on Animals (CCSEA) (Approval number: IISERK/IAEC/AP/2023/107).

Biosafety Clearance: The use of influenza type A/PR/8/1934 (H1N1) virus (ATCCVR95) (Ref. no: IISERK/IBSC/2019/005) and the HCoV‐OC43 (BEI Resources, NR‐52725) (Ref. no: IISERK/IBSC/2021/002) were approved by the Institutional Biosafety Committee (IBSC).

## Conflicts of Interest

The authors declare no conflicts of interest.

## Supporting Information

Additional supporting information can be found online in the Supporting Information section.

## Supporting information


**Supporting Information** Supplementary figures related to this article are available in the online version as a supplementary file.

## Data Availability

The data that support the findings of this study are available from the corresponding author upon reasonable request.
