# Supplementary material for: Antiviral Surface Protection of Polyhedral Oligomeric Silsesquioxanes (POSS)–Appended Hybrid Surface Materials
Source: Int J Biomater. 2026 Jun 19;2026:6543607. doi: 10.1155/ijbm/6543607 (PMC13282269; doi:10.1155/ijbm/6543607)
Supplement: Supplementary file 1 — Supporting Information Supplementary figures related to this article are available in the online version as a supplementary file. [file IJBM-2026-6543607-s001.docx]

*Supplementary Material*

**Antiviral Surface Protection of Polyhedral Oligomeric Silsesquioxanes (POSS)-Appended Hybrid Surface Materials**

Bibek Pramanik,^a^**^§^** Aparna Varma ^b^**^§^**, Chandan Kumar Pal ^a^, Jakir Ahmed^c^, Krishnendu Hazra ^a^, Totan Ghosh ^a^, Amirul Islam Mallick^b^* and Krishnendu Maji^a^*

^a^B. Pramanik, K. Hazra, T. Ghosh and K. Maji, Department of Applied Chemistry, Maulana Abul Kalam Azad University of Technology West Bengal, Nadia, West Bengal, 741249
E-mail: [krishnendu.maji@makautwb.ac.in](mailto:krishnendu.maji@makautwb.ac.in), [maji.krishnendu@gmail.com](mailto:maji.krishnendu@gmail.com)

^b^A. Varma and A. I. Mallick, Department of Biological Sciences, India Institute of Science Education and Research Kolkata, Mohanpur, Nadia, West Bengal, India-741246
^c^J. Ahmed Department of Chemical Sciences, India Institute of Science Education and Research Kolkata, Mohanpur, Nadia, West Bengal, India-741246.

Email: [amallick@iiserkol.ac.in](mailto:amallick@iiserkol.ac.in)

**Table of Content**

| **Content** | **Page No.** |
| --- | --- |
| Scheme S1 | 2 |
| Scheme S2 | 3 |
| Scheme S3 | 3 |
| Scheme S4 | 3 |
| Scheme S5 | 4 |
| Figure S1 | 4 |
| Figure S2 | 5 |
| Figure S3 | 5 |
| Figure S4 | 6 |
| Figure S5 | 6 |
| Figure S6 | 7 |
| Figure S7 | 7 |
| Figure S8 | 8 |
| Figure S9 | 8 |
| Figure S10 | 9 |
| Figure S11 | 9 |
| Figure S12 | 10 |
| Figure S13 | 10 |
| Figure S14 | 11 |
| Figure S15 | 11 |
| Figure S16 | 12 |
| Figure S17 | 12 |
| Figure S18 | 13 |
| Figure S19 | 13 |
| Cell Data | 14 |

Scheme S1: Synthesis of Compound 1 (POSS-Stearic Acid)

Scheme S2: Synthesis of POSS-Phe

Scheme S3: Synthesis of POSS-Leu

Scheme S4: Synthesis of Phe-Stearic Acid

Scheme S5: Synthesis of Leu-Stearic Acid


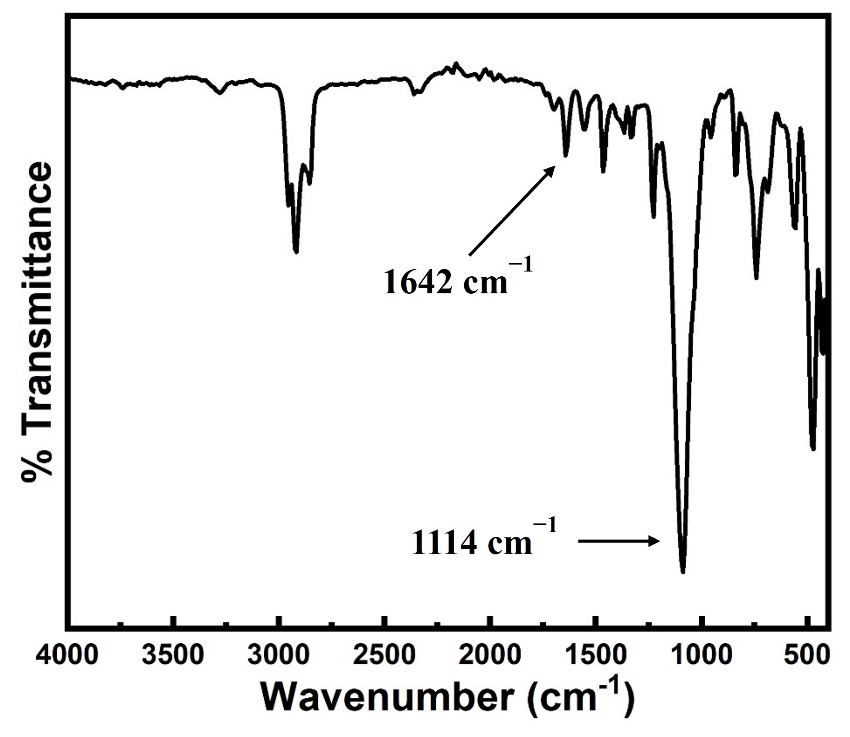


Figure S1. FTIR Spectra of Compound 1 (POSS-Stearic Acid)


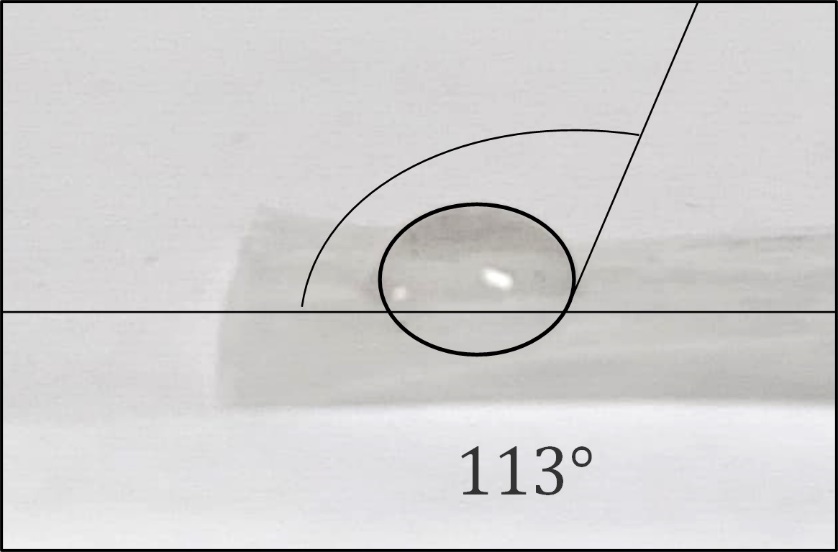


Figure S2. Contact angle measurement Experiment Compound 1(POSS-Stearic Acid)

Figure S3. ^1^H NMR Spectra of Compound 1 (POSS-Stearic Acid)

Figure S4: ^13^C NMR Spectra of Compound 1 (POSS-Stearic Acid)

Figure S5: ^29^Si NMR Spectra of Compound 1 (POSS-Stearic Acid)


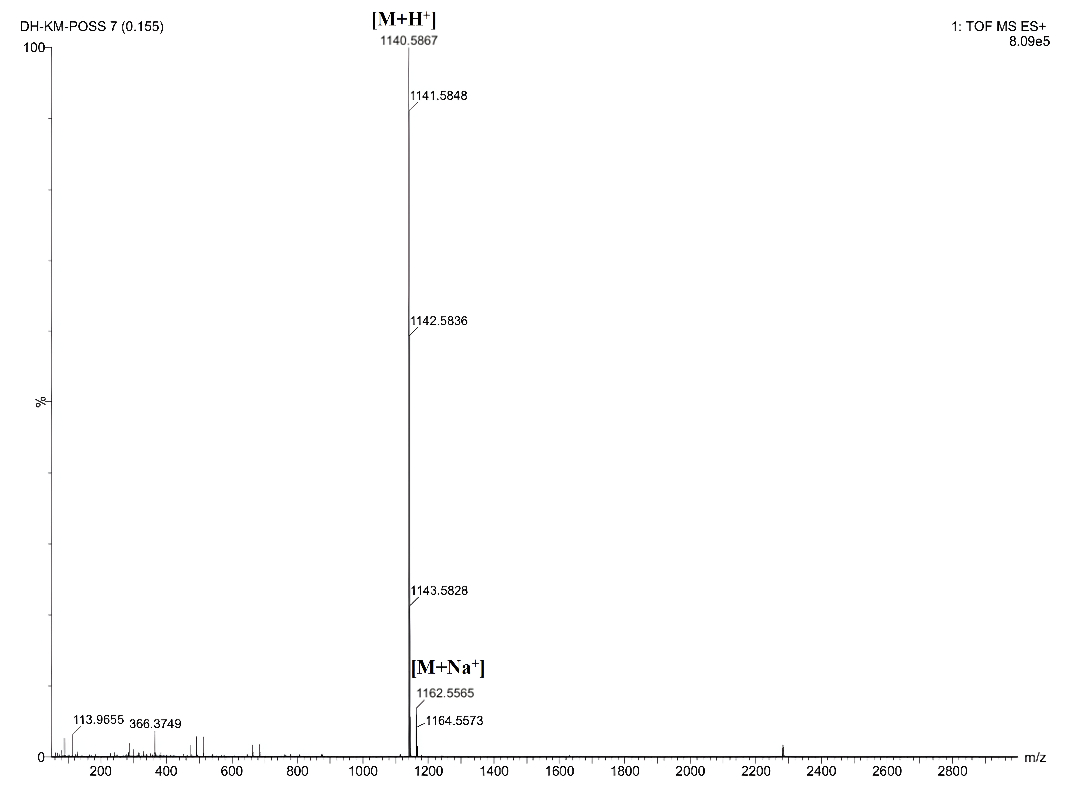


Figure S6: Mass Spectrometry Spectra of Compound 1(POSS-Stearic Acid)

. Figure S7: ^1^H NMR Spectra of POSS-Phe

Figure S8: ^13^C NMR Spectra of POSS-Phe

**
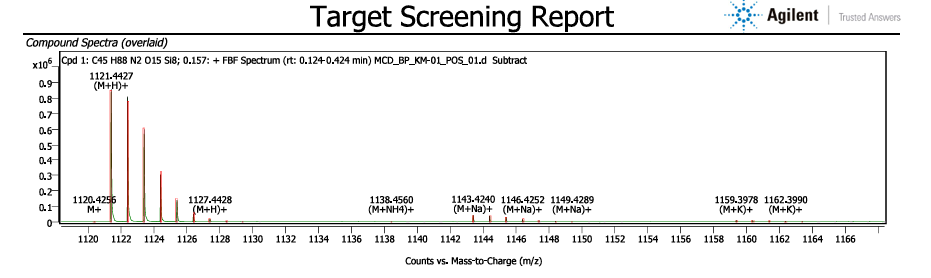
**

Figure S9. Mass Spectrometry of POSS-Phe

Figure S10: ^1^H NMR Spectra of POSS-Leu

Figure S11: ^13^C NMR Spectra of POSS-Leu


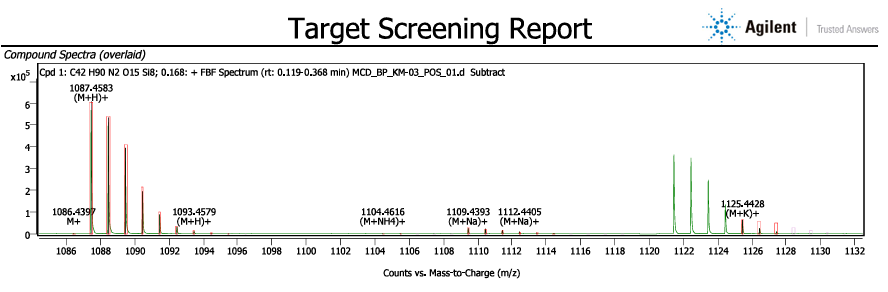


Figure S12: Mass Spectrometry data for POSS-Leu

Figure S13: ^1^H NMR Spectra of Phe-Stearic acid

Figure S14: ^13^C NMR Spectra of Phe-Stearic acid


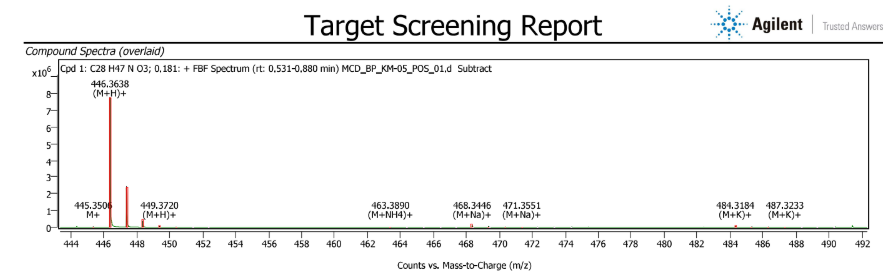


Figure S15: Mass Spectrometry data for Phe-Stearic acid

Figure S16: ^1^H NMR Spectra of Leu-Stearic acid

Figure S17: ^13^C NMR Spectra of Leu-Stearic acid


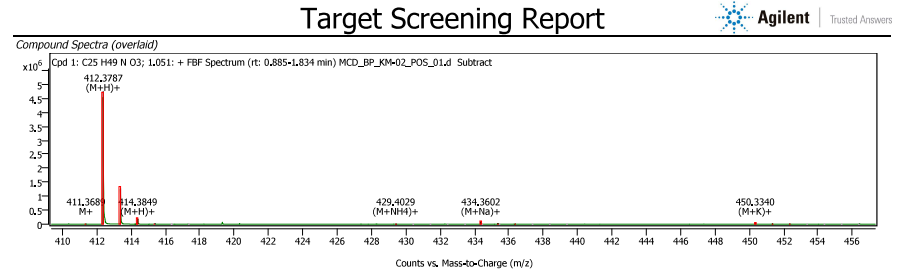


Figure S18: Mass Spectrometry data for Leu-Stearic acid


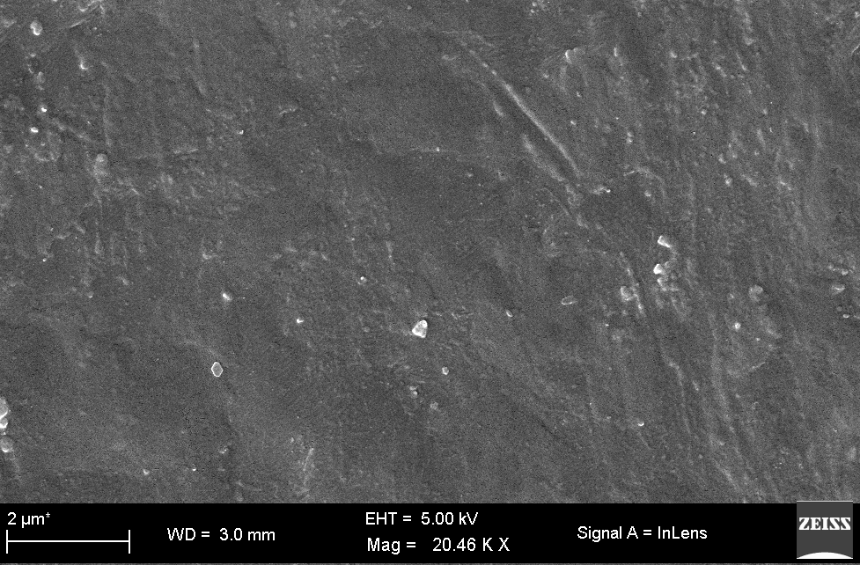


Figure S19: SEM Image of Coated Surface.

**Cell Lines Data**

**1. Species, Tissue of Origin, Official Cell Line Name:**

**MDCK Cells**

Species: *Canis lupus familiaris* (Dog)

Sex: Female

Tissue of origin: Kidney

Official cell line Name: Madin-Darby Canine Kidney (MDCK)

**Vero E6 Cells**

Species: *Chlorocebus sabaeus* (African Green Monkey)

Sex: Female

Tissue of origin: Kidney

Official cell line name: Vero E6 (Vero C1008)

**2. Source/Supplier and Date of Acquisition**

Both MDCK and Vero E6 cell lines were procured from the National Centre for Cell Science (NCCS) cell repository, Pune, India.

**3. Authentication Status**

Both cell lines were authenticated at the time of procurement from NCCS, Pune, India. The match profile showed a 100% match with the reference profile (1). As these cell lines were authenticated upon acquisition, no additional authentication was performed for this study

4. There are no reports of misidentification or contamination associated with this cell line.
